# Gender gaps in Australian research publishing, citation and co-authorship

**DOI:** 10.1007/s11192-023-04685-7

**Published:** 2023-03-21

**Authors:** Hamid R. Jamali, Alireza Abbasi

**Affiliations:** 1 School of Information and Communication Studies, Charles Sturt University, Wagga Wagga, NSW 2678 Australia; 2 School of Engineering and IT, The University of New South Wales (UNSW), Canberra, Australia

**Keywords:** Gender disparities, Disciplinary differences, Scientific publishing, Field of Research (FoR), Field Citation Ratio (FCR), Co-authorship, Australian science

## Abstract

Despite improvement in gender inequality in Australian science, the problem has not been fully addressed yet. To better understand the nature of gender inequality in Australian science, all gendered Australian first authored articles published between 2010 and 2020 and indexed in the Dimensions database were analysed. Field of Research (FoR) was used as the subject classification of articles and Field Citation Ratio (FCR) was used for citation comparison. Overall, the ratio of female to male first authored articles increased over the years, and this was true for all FoRs except for information and computing sciences. The ratio of single-authored articles by females was also improved over the study period. Females appeared to have a citation advantage, using Field Citation Ratio, over males in a few FoRs including mathematical sciences, chemical sciences, technology, built environment and design, studies in human society, law and legal studies, and studies in creative arts and writing. The average FCR for female first authored articles was greater than the average FCR for male first authored articles, including in a few fields such mathematical sciences where male authors outperformed females in terms of the number of articles.

## Introduction

Besides the fact that fairness and equal opportunity is everybody’s right, gender equality is good for innovation. Different forms of diversity in academia are regarded as a positive phenomenon that facilitates innovation (Hofstra et al., 2020; Nielsen et al., 2017; Page, 2009). However, gender disparities in different aspects of science are still widespread (Larivière et al., 2013) and there are country and disciplinary differences (Holman et al., 2018). The situation in Australia seems to be better than in some other countries. Gender has been in higher education discourse for some time (Gilbert et al., 2021). The Australian Government has a strategy to address gender disparity in STEM (Australian Government, 2021). Many Australian universities are acknowledged as “Employers of Choice for Gender Equality”, they promote gender pay equity, invest in gender diversity and parity, and are awarded Athena SWAN Accreditation which is an indication of their commitment to gender equality (Lipton, 2017; Manyweathers et al., 2020). Australian female scholars also seem to have citation advantages in some fields (Thelwall, 2020a, 2020b). However, there is still a lot to do to achieve reasonable gender equality. Some argue that institutional policies and Athena SWAN Accreditation haven’t achieved a lot in terms of meaningfully addressing gender inequality in academy (Gilbert et al., 2021; Ovseiko et al., 2017). Australian Higher Education Statistics, for instance, still shows that while overall the number of female academics is more than males (between 56 to 59 per from 2017 to 2021), the number of senior male academics (above senior lecturers) is twice the number of female senior academics (DESE, 2021), which can be an indication of the glass ceiling. A higher representation of female academics in the workforce does not translate to equality in other aspects such as productivity, impact, and leadership. This is the case for some other countries such as Russia too where 60% of academics are female but there are large gender gaps in authorship and impact (Pilkina & Lovakov, 2022). To properly address gender inequality and to form better policies and implement them, the nature and extent of gender disparity need to be better understood.

This study aims to investigate the gender disparity in Australian research by studying publication productivity, citation impact and co-authorship pattern of Australian first authored articles between 2010 and 2020. Unlike some previous studies, this study uses the Dimensions database for its broader coverage and the ANZSRC Field of Research (Australian Government, 2008), which is the base in the Australian national research evaluation, for the subject classification of articles. The research specifically answers the following questions:

- What is the ratio of female first authored articles by the Field of Research from 2010 to 2020?
- What is the ratio of single-authored articles for male and female authors by the Field of Research?
- Are there any differences in citation impact of female first and male first authored articles in different Fields of Research?

## Literature review

Researchers have investigated many aspects of research from a gender perspective over the last decade or so. Here we review some recent relevant studies in relation to authorship, co-authorship and citation, the three areas that we investigated.

### Authorship

Studies on authorship and representation of female authors generally show under-representation of females in most fields and this seems to be a global trend, although there are country differences. A large-scale study of 36 million authors from Science, Technology, Engineering, Mathematics, and Medicine (STEMM) for 15 years showed that the gender gap, despite progress, will probably persist for a long time, especially in physics and maths, computer science and surgery. The gap is much larger for senior academic positions, and publications in prestigious journals. Rich countries such as Switzerland, Germany and Japan have fewer female authors compared to poorer countries (Holman et al., 2018). In India, an analysis of 2008–2017 for 50 most productive institutions showed that the ratio of female first authored papers to male first authored papers varied by discipline and was highest in biology, agriculture, and medical sciences and lowest in engineering and information technology (Paswan & Singh, 2020). Another study of articles from India published in 2017 showed that in all 26 broad fields, the ratio of male first authors to female first author was at least 1.5, and the overall ratio was 2.8 male first authors to female first author (Thelwall et al., 2019a). In the UK, the data for 2017 showed significant differences in gender gaps for broad fields. While the number of female-first authored papers in veterinary science and nursing was more than twice that of male first authored papers, in mathematics and physics there were more than three times male first authored papers than female first authored papers (Thelwall et al., 2020). Analysing 30 million articles published between 2014 and 2018 from 27 broad fields from 31 countries with the most articles in Scopus, Thelwall and Mas-Bleda (2020) found that in most countries half of the subjects had more than average males (e.g., maths) or more females (e.g., immunology and microbiology). They also found that countries with high proportion of female authors also had higher gender disparity. A large longitudinal study found that while there has been an increase in the female involvements in science over the last 60 years, gender gaps in research productivity and impact have also increased (Huang et al., 2020). Studies of specific disciplines such as physicians (Eloy et al., 2013), family medicine in Switzerland (Sebo et al., 2021), cardiology in Australia (Segan & Castles, 2019) and geoscience (Pico et al., 2020) also reveal under-representation of female authors. A recent study of authorship showed that the disparity was worsened (at least in some fields) during the COVID-19 pandemic as the share of female first authors in biomedical fields decreased on average by about 9% across fields (Muric et al., 2021).

### Co-authorship

Past studies have shed some light on the gender aspect of collaboration and contribution. Their results are not always consistent and there are certainly some disciplinary differences. A large-scale study of eight million papers (JSTOR corpus) showed that in specific fields, there was a significant underrepresentation of female authors in single-authored articles, and male authors dominated prestigious first and last authorship positions (West et al., 2013). A study of US researchers in STEM showed that females compared to males have notably fewer distinct co-authors over the course of their careers, however, this difference could be explained by lower publication rate and shorter career lengths of females. The study also reported that females are less likely to co-publish with their previous co-authors (Zeng et al., 2016). A large-scale analysis in mathematics showed that although female mathematicians number has increased three times since 1970, they publish less than males at the beginning of their careers, and they are more likely than men to leave academia, they are less representative in top-ranked journals, and they published fewer single-authored papers (Mihaljević-Brandt et al., 2016). A study of more than 270,000 scientists showed that men were more likely to collaborate with men, and women are more egalitarian. The only exception was in engineering field where the gender gap diminished with the increasing number of collaborators (Araujo et al., 2017). In computer science, successful male and female scientists have similar collaboration patterns, collaborating with more colleagues and establishing more repetitive collaborations that last longer. However, women on average are more likely to be gender homophile in their collaboration and to leave academia in all career ages (Jadidi et al., 2018). In terms of contribution, an analysis of author contribution notes of *PLoS* articles between 2008 and 2012 showed that gender played an important role in authorship roles and tasks with female authors being more likely to do experiments, and male authors were more likely to be involved in all other tasks (Macaluso et al., 2016).

### Citation impact

A few studies that compared the citation impact of male and female authors have found some superiority for female authors. A study of one university in Croatia showed that female STEM full professors were significantly more cited than male colleagues (Wild et al., 2020). Two studies on six-large English-speaking countries (i.e., the UK, Ireland, the USA, Canada, Australia, and New Zealand) by Thelwall (2020a, 2020b) found some citation superiority for female authors. Thelwall’s (2020b) analysis of male and female first authored research for 27 broad fields (1996 to 2014) revealed that overall female-first authored papers had a citation advantage in some years. There were differences based on countries including citation advantage for Canadian male authors for most years, and Australian female first authored medicine publications attracted more citations for all years (Thelwall, 2020b). No general pattern for gender gap changes over time could be seen. Another study for the period 1996–2018 for seven English-speaking countries (the above six plus Jamaica) found that in all countries except the USA, female authors had a small citation advantage over male authors. This advantage was largest for the British and Australian female authors (Thelwall, 2020a).

Overall, the literature review shows that while there has been progress in some areas, such as representation in the workforce and publications, there are still gender gaps in all aspects of science, including productivity and impact. These gaps vary by disciplinary and country. However, the evidence is yet fragmented and more research is required to understand the nature and extent of gender gaps in the context of each country and discipline to be able to find the root causes and address them. Our study, therefore, focuses on Australian science and uses a discipline classification that is specifically used in research policy and management in Australia so the results can potentially better inform policies and initiatives in this area.

## Methods

### Data

The dataset in this research consists of records for all document type ‘article’ in the Dimensions database that were published from 2010 to 2020 and had a first author with an Australian affiliation and a first name from which a gender could be inferred with high accuracy. The eleven years was chosen as it provided a long enough period to study both the current status quo and any trend in recent years. The Dimensions database was chosen (over Web of Science and Scopus) as it has a more exhaustive coverage of journals (Singh et al., 2021) and provides field normalised citation data as well as Field of Research (FOR) codes based on the Australian and New Zealand Standard Research Classification (ANZSRC) (Australian Government, 2008), which comprises a list of hierarchical FoR Codes for articles. It has a hierarchy of three levels, including 22 Divisions, 157 Groups and 1238 Fields. Dimensions provides FoR codes for each article at Groups level. The codes are assigned at the article level and unlike other classification systems used in Scopus or Web of Science, they are not based on journal classification. FoR is important in the Australian context as it is the classification used in the national research evaluation system called Excellence in Research for Australia (ERA).

To obtain and clean the data, a search was conducted in August 2021 for all records with at least one author affiliated with an Australian institution. This included 913,463 records. Dimensions increased the download limit for one of the researchers so records could be downloaded in batches of 50k records. After extracting the meta-data of all the 913,463 records, 5932 records that were found to be corrections, retraction notes, editorials and so on were removed from the dataset. This was done because Dimensions publication type includes only six categories: article, book, chapter, monograph, preprint, and proceeding. Article is defined as “Article from a scientific journal or trade magazine, including news and editorial content”, and to keep only peer-reviewed articles, we removed other types.

Then all records whose first author had Australian affiliation were separated. Because Dimensions affiliation information is not very structured, we used python codes to clean affiliation information and identify those with Australian affiliation. This resulted in 614,368 records. Out of those, we found 75,377 records with incomplete first author information, including not having a full name (i.e., only initials), which were removed and left us with 538,991 records. Similar to most of past studies we used only the first author for the analysis. This could impact the results if authors are listed in alphabetical order, but past research shows that the alphabetisation has limited impact on the assumption that the first author gender is the main author gender (Thelwall et al., 2020).

### Gender detection

For gender detection, we used 1021 male and 3937 female names, extracted from the USA 1990 census. We only used names that had been used at least 90% of the time by the same gender. This data has been used by Thelwall in the study of the UK, USA, India, and a few other countries (Thelwall, 2018; Thelwall et al., 2019a, 2019b, 2020). This resulted in the gender identification for 402,511 names (74.7% of articles) in our dataset.

To check the accuracy of gender detection, we drew a random sample of 1018 Australian first authored articles from the entire dataset after removing those without a first name (initials etc.) with a random number generator. Then we manually assigned gender to them by searching for their homepage online and identifying a picture or a gendered pronoun where possible. The extra 18 was because for 18 authors we could not find any information to assign gender. Then we compared this set with the outcome of the automatic gender assignment. The automatic method correctly identified the gender of 390 out of 446 female authors in the set, hence a recall of 87.4% and it misidentified 2 males as females, hence a precision of 99.5%. the method also correctly identified the gender of 427 out of 543 male authors, hence a recall of 78.6% and the precision was 100% as not female was identified as male.

### Limitation

There are known issues associated with using bibliometrics for gender studies of science that have been discussed in the literature (see for instance Mihaljević-Brandt et al., 2016). Some of the issues are related to the use of the first name for gender detection and the data sources used for gender identification. Using the first name has the issue of unisex names. The data sources and services used for gender identification are still evolving but they are mostly based on data from Western countries, particularly the USA. This can create a bias as the social security or census data of the USA might not well represent populations from other parts of the world and their names. Both Australia and the USA have a lot of migrants, but the composition of these migrants might be different and therefore names registered in the USA census might not be the best source of data for people from a non-English culture in Australia. However, this is a limitation as there is no reliable and comprehensive data from Australia for name identification. Other commercial services such as GenderAPI are not usually used in research for lack of transparency and reliance on social media for gender identification (Thelwall et al., 2020). The limitations of name-to-gender online services including GenderAPI, NameAPI, genderize.io, NamSor, and python-package gender-guesser are also discussed in Santamaría and Mihaljević (2018) in a comparitive study.

## Results

### Gender gap in authorship

**Fig. 1:**
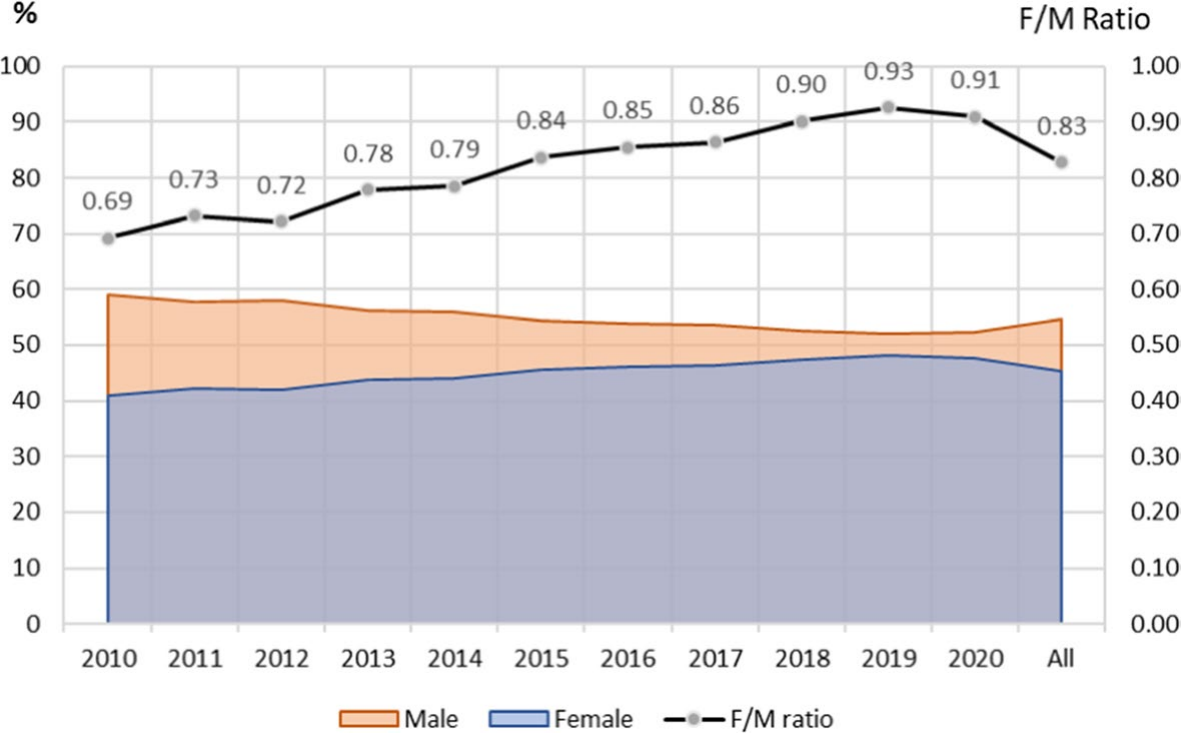
Percentage of articles by the gender of the first author by year and F/M ratio

Comparing the ratio of female to male first authored articles over the years, Fig. 1 shows that the ratio has almost steadily increased from 0.69 in 2010 to 0.91 in 2020. The value in 2020 slightly decreased compared to 2019 and it is unknown to us whether this was due to the COVID pandemic or not. However, the overall ratio for all years is 0.83, which means females’ share is not equal to males. In total, there were 182,303 female first authored articles compared to 220,208 male first authored articles.

Table 1 presents the ratio of female to male first authored articles per year for each Field of Research (FOR) code, with the overall ratio for all FoRs between 2010 and 2020 in the last column. The fields shown in bold (and cells with grey shading) have an F/M ratio of above 1 which means they have more female first authored articles than male first authored articles. They include medical & health sciences (11), education (13), studies in human society (16), psychology and cognitive sciences (17), studies in creative arts and writing (19), language, communication and culture (20). On the other hand, the ratio is less than a third in mathematical sciences (01), physical sciences (02) and engineering (09), and less than half in several fields including chemical sciences (03), earth sciences (04), information and computing sciences (08) and technology (10). Analysing the F/M ratio over time reflects an increase between 2010 and 2020 for almost all the fields including natural sciences where males dominate. Information & computing science (08) is the only field with more volatility and an overall decline in the F/M ratio between 2010 and 2020.

**Table 1:**
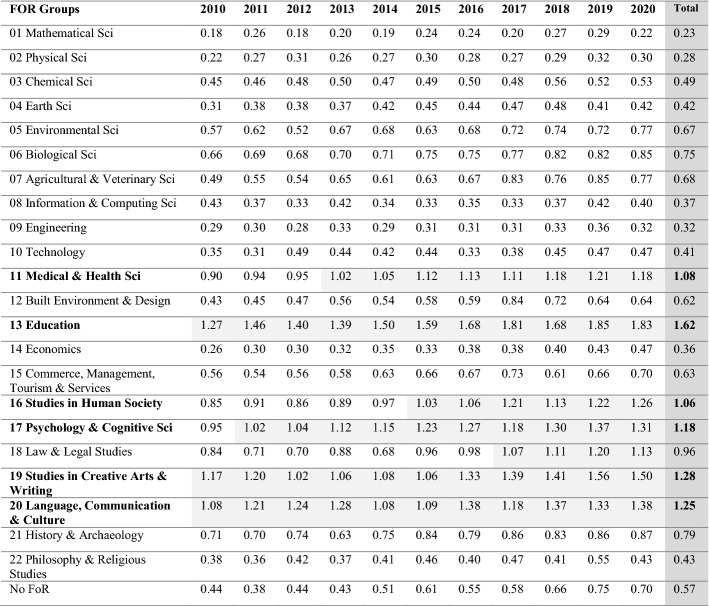
F/M ratio by year by Field of Research

### Co-authorship patterns

**Fig. 2:**
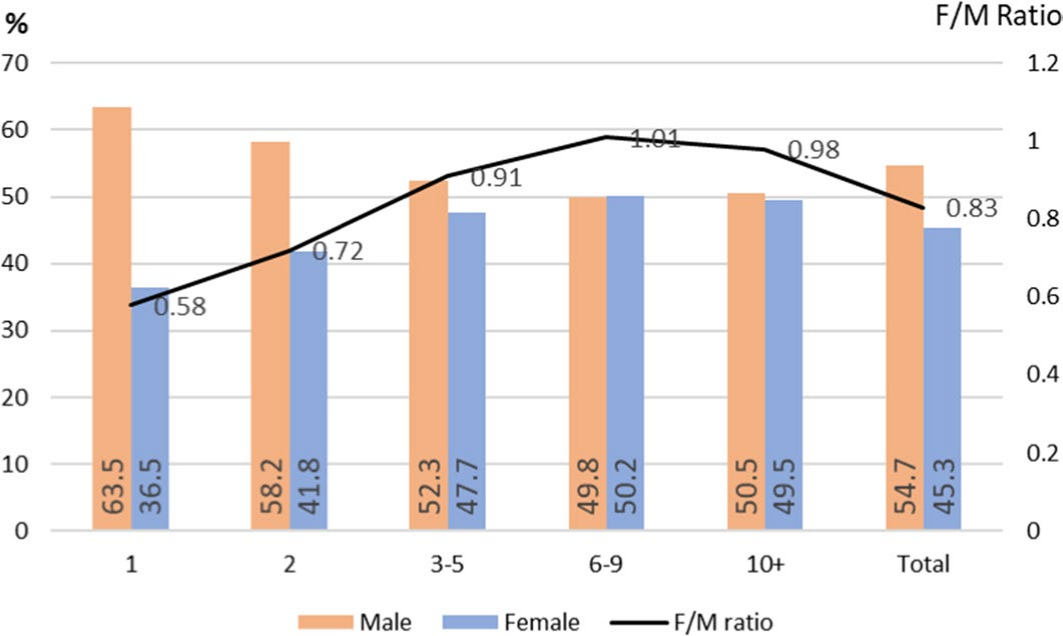
Percentage of articles by the number of authors and F/M ratio

**Fig. 3:**
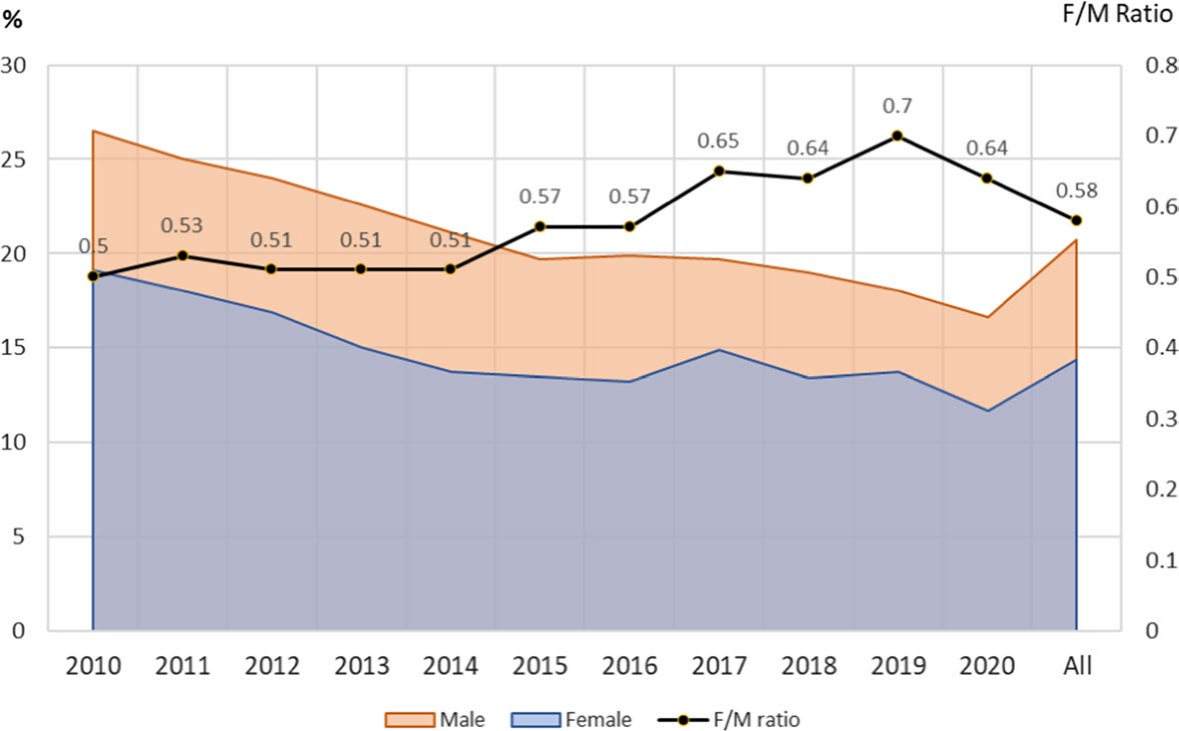
Percentage of single-authored articles by gender per year and F/M ratio

Comparing co-authorship patterns (Fig. 2), overall, females were more likely to be the first author of multi-author articles than single-authored articles. The first author of only 36.5% of single-authored articles was female while that figure for articles with 6 to 9 authors was around 50%. Over the years (Fig. 3), the percentage of single-authored articles by females has increased as you can see in the F/M ratio. The percentages in Fig. 3 are the percentages of single-authored articles out of the total publications by each gender in that year. For instance, in 2020, 19.1% of all female first authored articles were single-authored articles. That percentage has declined for both genders during the study period simply because we know the rate of collaboration has increased over the years globally in all fields (Fanelli & Larivière, 2016) including social sciences (Henriksen, 2016).

Table 2 shows the female to male ratio for single-authored articles by the Field of Research by year. Only in three disciplines, the ratio was high for most years. They were education; studies in creative arts and writing; and language, communication and culture. These disciplines were also among the disciplines that had a higher F/M ratio overall for all articles. The ratio was more volatile in some fields such as physical sciences, information and computing sciences, and engineering than in some other fields.

**Table 2:**
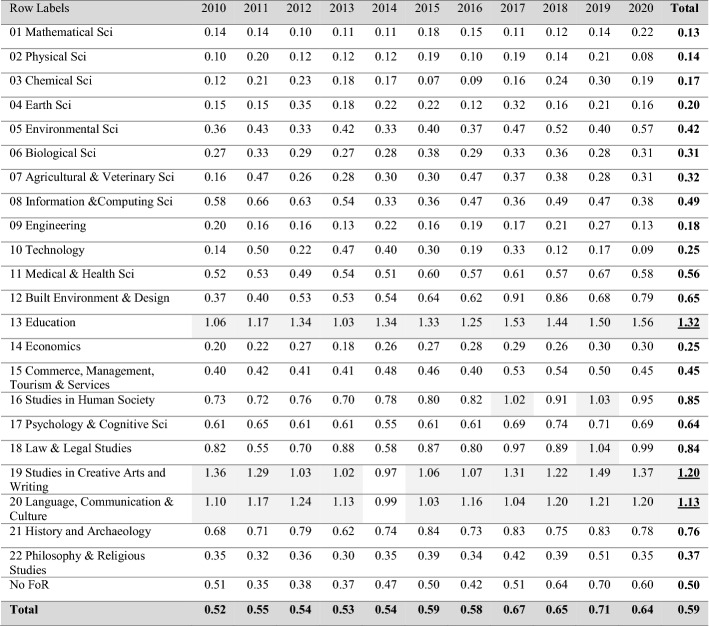
F/M ratio of single-authored articles by Field of Research per year

### Citation impact of male and female authors

We used Field Citation Ratio (FCR) for comparing citation impact. As defined by Dimensions, FCR as a citation-based measure of articles’ scientific influence is calculated by “dividing the number of citations [an article] has received by the average number received by documents published in the same year and the same Fields of Research (FoR) category” (Dimensions, 2019). As explained by Dimensions “The FCR is calculated for all publications in Dimensions that are at least 2 years old and were published in 2000 or later. Values are centred around 1.0 so that a publication with an FCR of 1.0 has received the same number of citations as the average, while an article with an FCR of 2.0 has received twice as many citations as the average for the Fields of Research code(s)” (Dimensions, 2019).

**Fig. 4:**
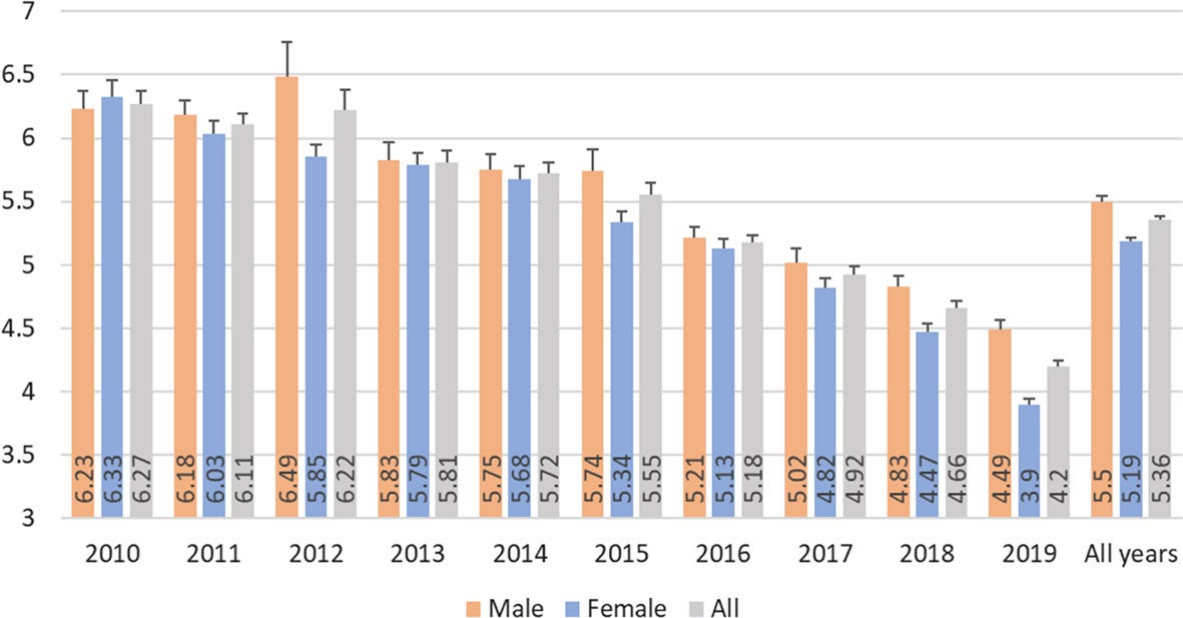
Average FCR by gender per year (with standard error bars)

As Fig. 4 shows the average FCR for males was greater than that of females for all years except 2010. An independent sample *t* test (*t* = 6.2, *p* < 0.001) showed the mean difference between male and female was overall statistically significant but the effect size was very small (*d* = 0.021). When looking at the difference between female to male FCR (Female FCR–Male FCR) for individual FoRs over the years (Table 3), we see that for some fields and some years (shaded in grey) female FCR is greater than that of males. For instance, female FCR in chemical sciences in 2010 was 2.53 greater than male FCR in that FoC in that year. Looking at the overall value for all years (last column), we see that in mathematical sciences (01), chemical sciences (03), technology (10), built environment and design (12), studies in human society (16), law and legal studies (18), and studies in creative arts and writing (19) the average FCR for female first authored articles is greater than the average FCR for male first authored articles. These are not necessarily the field where there were more female first authored articles (bold FoRs in Table 1), and in some cases, it is the opposite. Some of these such as mathematical sciences are fields where male authors outperformed females in terms of the number of articles. This indicates that while there were fewer female first authored articles, on average they have received more citations.

**Table 3:**
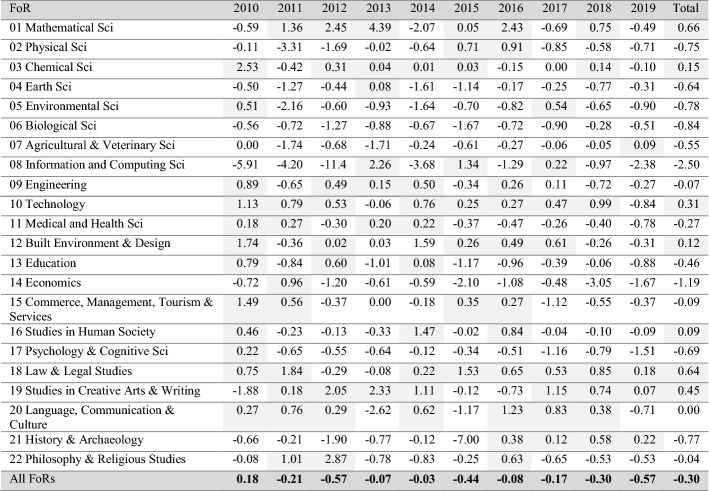
Difference between average female FCR and male FCR (female minus male) by Field of Research per year

## Discussions

We used Dimensions data to analyse the gender distribution of articles whose first authors had an Australian affiliated between 2010 and 2020. The choice of database, as is the case with any database, imposed some limitations on the study. Document type and affiliation data in Dimensions is not as structured and clean as such data one can obtain from Scopus or Web of Science. Although we thoroughly cleaned the data before analysis, these issues might have had a marginal impact on the data. However, Dimensions had the advantage of providing Field of Research at the article level which is important in the Australian context. Moreover, it provides field normalised citation that was used for citation impact comparison. The gender detection method also creates limitations as it is a binary and non-inclusive method and relies on data (e.g., US Census) that might not necessarily well reflect the demographic of Australia.

The study showed that gender parity in article authorship in Australia has improved over the last decade. This is in line with the findings of previous studies. Thelwall (2020b) also maintained that Australia is approaching gender parity as the percentage of female first authored articles increased from about 42% in 2010 to about 47% in 2018. Although our data show that the ratio of female to male first authored articles increased over the years, there is still work to do before females have an equal share of the articles. The ratio was the lowest in Science, Technology, Engineering and Mathematics (STEM). But a more concerning fact is that the rate of progress in these fields was also slow. For instance, while in agricultural and veterinary sciences F/M ratio increased from 0.49 in 2020 to 0.68 in 2020, in physical sciences it only increased from 0.22 to 0.28. Holman et al. (2018) study showed that it will be a few more decades before male and female shares are equal in Australia in all fields. Various factors might explain a lower rate of authorship for female authors in some fields. For instance, past research has shown that at least in some fields female authors might have different publication patterns, publishing more book chapters and fewer articles in prestigious journals (Mayer & Rathmann, 2018).

The data showed the usual global trend of increasing co-authorship rates (Fanelli & Larivière, 2016) for both males and females. However, the number of solo female authored articles increased over the study period which is a sign of improvement. However, the F/M ratio for single-authored articles was worse than the overall F/M ratio and some fields that had a high overall F/M ratio such as medical and health sciences, and psychology and cognitive sciences did not have similar F/M ratio for single-authored articles. It might be that collaboration is more common in these fields. However, it is not clear whether the lower F/M ratio for single-authored articles in many fields is a result of the preference of female authors to collaborate instead of writing solo articles or whether there is a hindrance in those fields that makes it more difficult for female authors to write by themselves. Past research showed that the rate of solo publishing is affected by various factors such as team size and discipline (e.g. fewer solo papers in STEM) among other things (Kwiek & Roszka, 2022).

Using mean normalised log citation score (MNLCS), Thelwall (2020b) found a citation advantage for female first authored Australian articles (based on Scopus data). This wasn’t the case for the FCR data in this study. Australian male authors had a citation advantage overall in most years. However, several Fields of Research, including a few fields that are male dominated such as mathematical and chemical sciences, showed citation advantages for female authors. This difference between Thelwall’s study and ours might be explained by the data used. The Dimensions database includes more outlets than Scopus (Singh et al., 2021) and its content probably has a different disciplinary distribution. Moreover, FCR was only available for 327,171 (81.3%) of articles. It is calculated when there are at least 500 articles in a FoR code in a given year, and for articles that have at least one 4-digit FoR code, which is a limitation. The number of articles that had FoR codes was 395,825 (98.4% of articles). Moreover, FoR in Dimensions is assigned at the article level based on the topic of articles, while the Scopus classification used in the study by Thelwall is a journal classification.

Citation advantage is not linked to a gender’s dominance in terms of the number of publications. Australian females had citation advantages in fields that were male dominated including some of the STEM files such as mathematics and chemistry. This might be because those female scholars who enter these fields might be passionate about their research as they go against the odd into fields that are not probably as female-friendly as some other fields and therefore, they simply do better quality research for they are more passionate and dedicated, or perhaps they research things that are more useful (Thelwall, 2020a). Enrolment and completion data from universities indicates that most women who enter into fields such as mathematics complete their courses (DESE, 2021) which can be another indication of their interest, dedication and passion. However, there are factors that cause fewer female STEM researchers stay in their fields. In the case of astronomy in Australia, women depart astronomy at two to three times the rate of men (Kewley, 2021) and this results in underrepresentation.

## Conclusions

The results show an overall improvement trend in terms of the total ratio of female to male first authored articles and this trend is true for most fields of research, although the rate of improvement is not the same in all fields and generally the STEM fields have a slower increase. Moreover, the percentage of single-authored articles by females has also increased over the years which is another improvement. In terms of citation advantage, however, there is no clear trend. There are still wide disciplinary differences and overall, there is more work to do especially in the STEM area to achieve optimal parity in terms of participation in disciplines. More research needs to be done on the nature of some differences such as the ratio of single-authored papers and the reasons for gender-related citation advantage in certain fields.
